# Supplementary material for: 1,10-phenanthroline inhibits sumoylation and reveals that yeast SUMO modifications are highly transient
Source: EMBO Rep. 2024 Jan 5;25(1):68–81. doi: 10.1038/s44319-023-00010-8 (PMC10897377; doi:10.1038/s44319-023-00010-8)

APPENDIX FIGURE

1,10-phenanthroline inhibits sumoylation and reveals that yeast SUMO modifications are highly transient

by

J. Bryan McNeil, Su-Kyong Lee, Anna Oliinyk, Sehaj Raina, Jyoti Garg, Marjan Moallem,

Verne Urquhart-Cox, Jeffrey Fillingham, Peter Cheung, and Emanuel Rosonina

Contents

Appendix Figure S1 Figure Legend ………………………… p. 2

Appendix Figure S1 ……………………………………….. p. 4

Appendix Figure S1. Supporting data

A. 1,10-phenanthroline inhibits in vitro sumoylation with SUMO1. In vitro sumoylation reactions were set up using the SUMO1 isoform, and including DMSO or 10, 15, or 20 mM of 1,10-phenanthroline. "DMSO –Mg/ATP" refers to a negative control reaction in which $MgCl_2$ and ATP were excluded. Reactions were analyzed by immunoblot using the SUMO1 antibody.

B. Limiting magnesium in vivo does not significantly affect sensitivity to 1,10-phenanthroline. Samples of yeast lysates were prepared from wild-type yeast (W303a) grown to mid-log phase in synthetic complete (SC) medium prepared using yeast nitrogen base (YNB) lacking $MgSO_4$, but supplemented with either 0.5 g/L (1X) or 0.05 g/L (0.1X) of $MgSO_4$. Both samples were treated with the indicated concentrations of 1,10-phenanthroline (ranging from 0 to 500 μg/mL) for 30 min. Lysates were prepared, yields determined by absorbance measurements at 280 nm, and equal amounts were analyzed by SDS-PAGE and SUMO immunoblot.

C. Treatment of yeast cultures with zinc chelator TPEN does not significantly affect global sumoylation levels. Samples of yeast lysates were prepared from wild-type yeast (W303a) grown to mid-log phase in YPD, then treated with TPEN (*N*,*N*,*N'*,*N'*-tetrakis(2-pyridylmethyl)-1,2-ethanediamine) at the concentrations indicated for 30 min. Lysates were prepared, yields determined by absorbance measurements at 280 nm, and equal amounts were analyzed by SDS-PAGE and SUMO immunoblot.

D, E. Effects of 1,10-phenanthroline on sumoylation in human and *Tetrahymena* cells. Cultures of human cell lines 293T and HeLa, and a strain of *Tetrahymena thermophila*, were grown and treated with the indicated amount of 1,10-phenanthroline for the indicated durations. Lysates were then prepared and analyzed by immunoblot with the indicated antibodies.

F. 1,10-phenanthroline does not elevate Ulp1 desumoylation activity in vitro. Samples of yeast lysate from the *ulp1-1* strain were treated with either 0.01 or 0.001 units of purified Ulp1 protein (Abbkine), according to the supplier's instructions, or left untreated. Included in the reaction mixes were the indicated concentrations of 1,10-phenanthroline (0 to 50 mM) and reactions were incubated for 30 min at 30°C. After incubation, an equal volume of SDS-PAGE sample buffer was added, and reactions were assayed by a SUMO immunoblot.

G. Rapamycin does not reduce SUMO conjugation levels in the Anchor-away parent strain. Cultures of the strain were treated with rapamycin for the indicated durations, then lysates were prepared and analyzed by SUMO and GAPDH immunoblots.

Appendix Figure S1

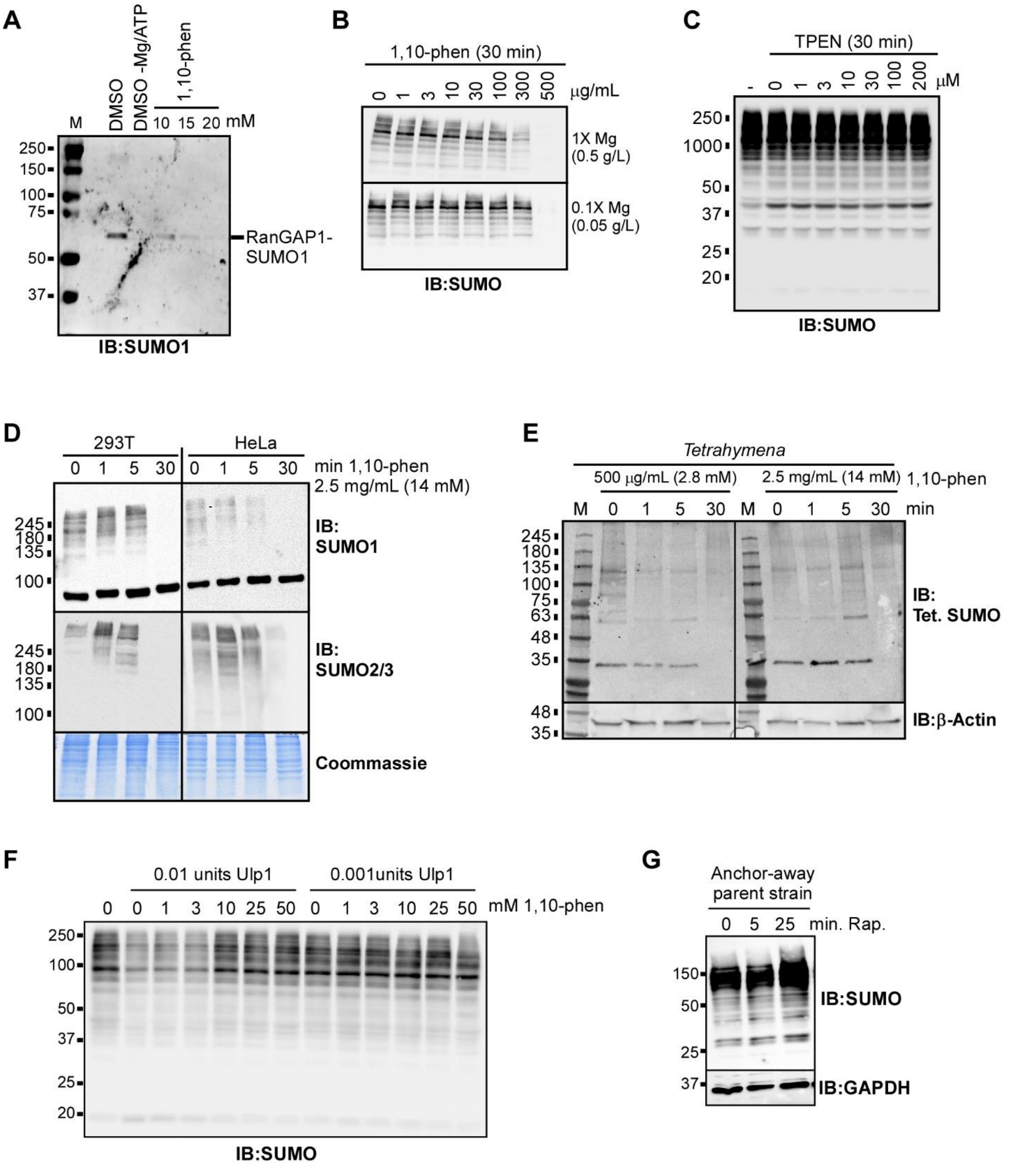

Supplement: Supplementary file 1 — Appendix [file 44319_2023_10_MOESM1_ESM.pdf]
