# Supplementary figures and images for: 1,10-phenanthroline inhibits sumoylation and reveals that yeast SUMO modifications are highly transient
Source: EMBO Rep. 2024 Jan 5;25(1):68–81. doi: 10.1038/s44319-023-00010-8 (PMC10897377; doi:10.1038/s44319-023-00010-8)

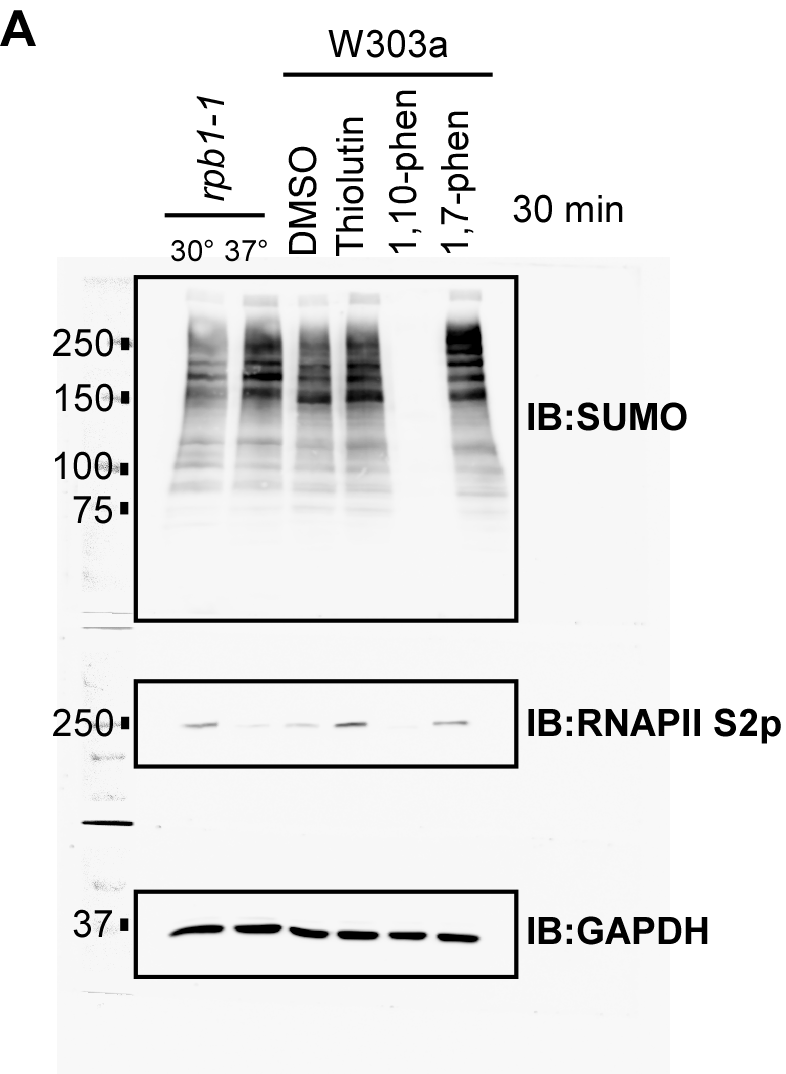

Supplement: Supplementary file 3 — Source Data Fig. 1 [file 44319_2023_10_MOESM3_ESM.zip › Figure 1/1A.tif]

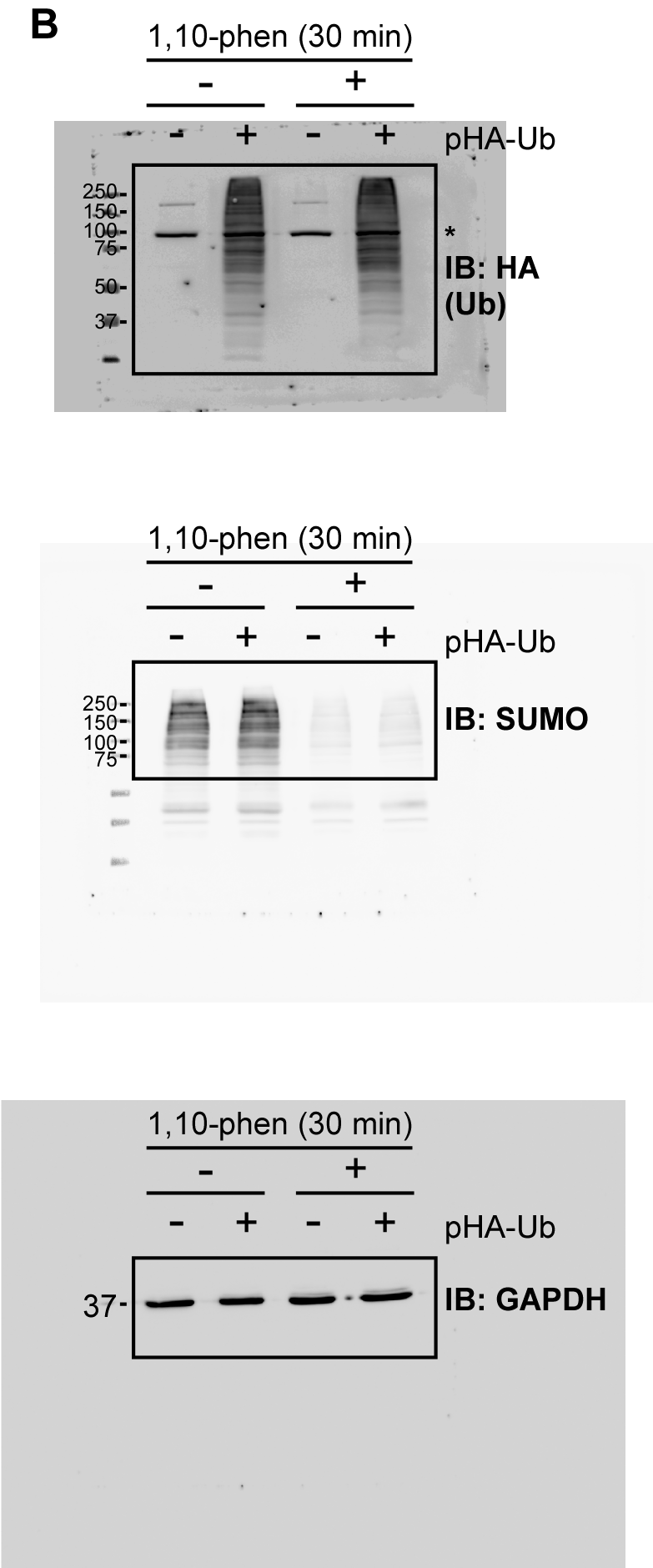

Supplement: Supplementary file 3 — Source Data Fig. 1 [file 44319_2023_10_MOESM3_ESM.zip › Figure 1/1B.tif]

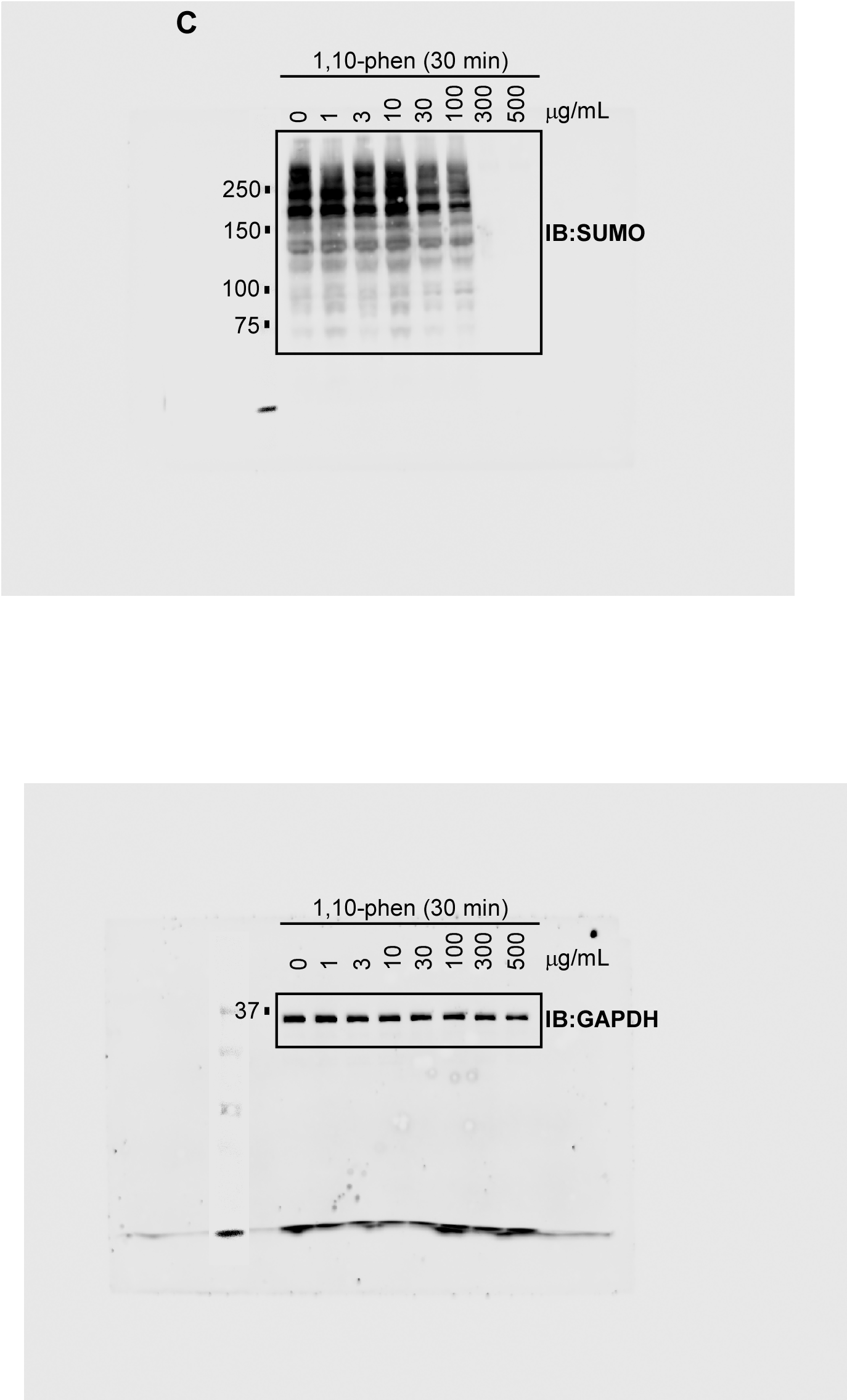

Supplement: Supplementary file 3 — Source Data Fig. 1 [file 44319_2023_10_MOESM3_ESM.zip › Figure 1/1C.tif]

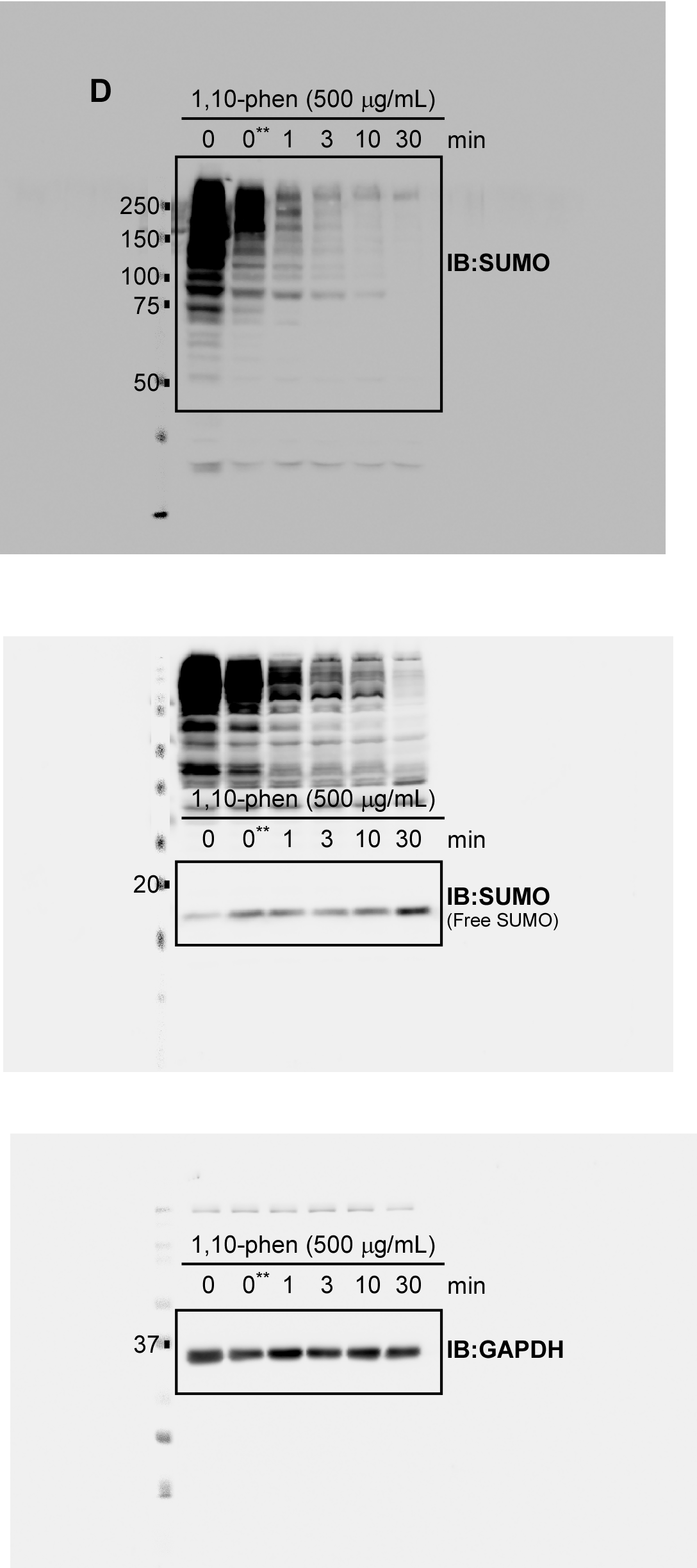

Supplement: Supplementary file 3 — Source Data Fig. 1 [file 44319_2023_10_MOESM3_ESM.zip › Figure 1/1D.tif]

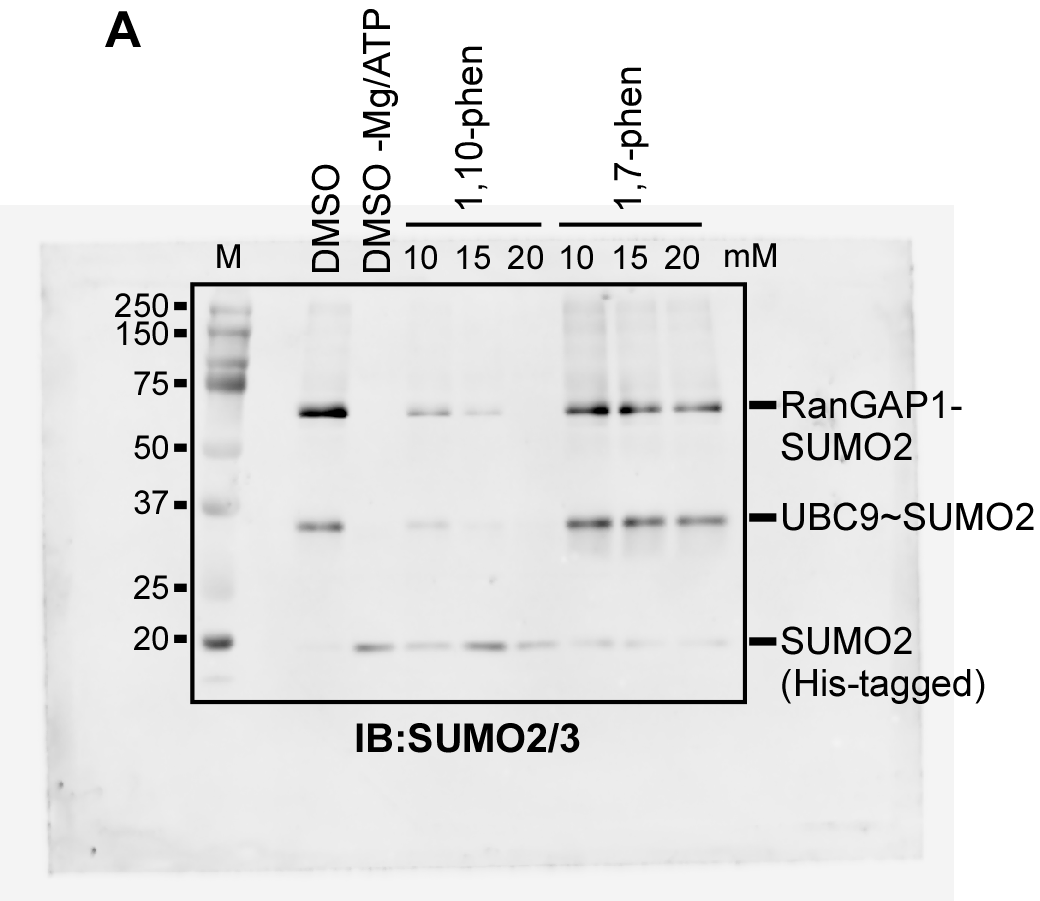

Supplement: Supplementary file 4 — Source Data Fig. 2 [file 44319_2023_10_MOESM4_ESM.zip › Figure 2/2A.tif]

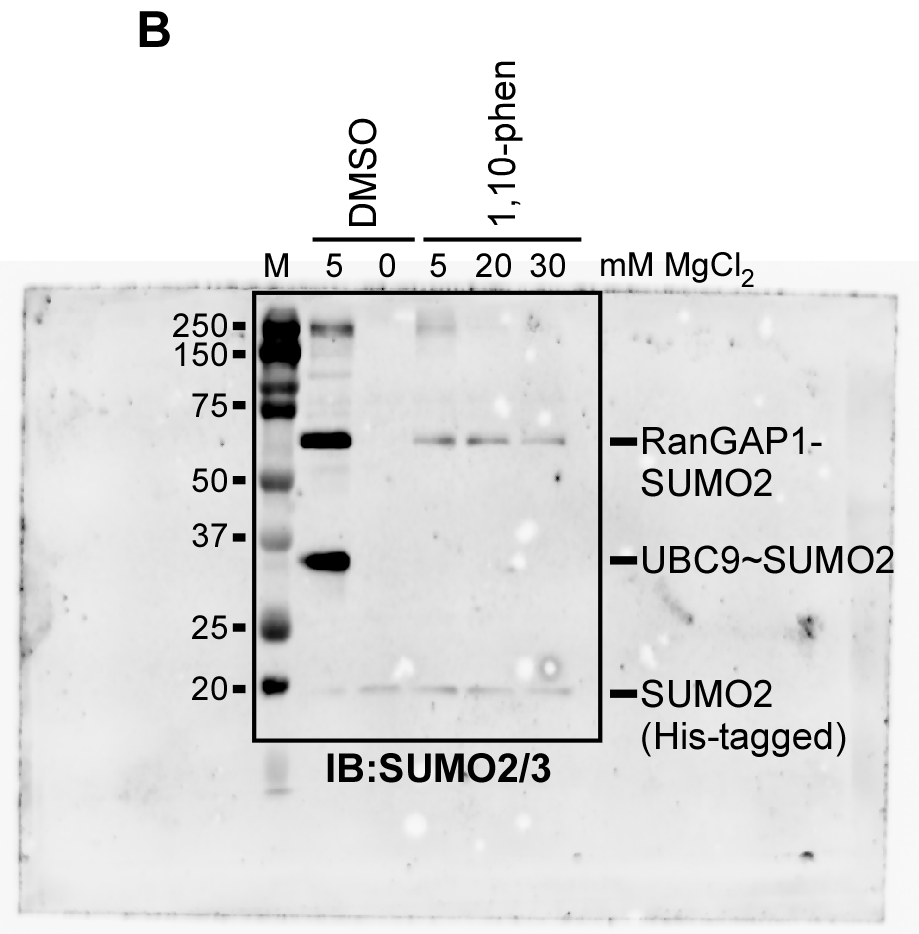

Supplement: Supplementary file 4 — Source Data Fig. 2 [file 44319_2023_10_MOESM4_ESM.zip › Figure 2/2B.tif]

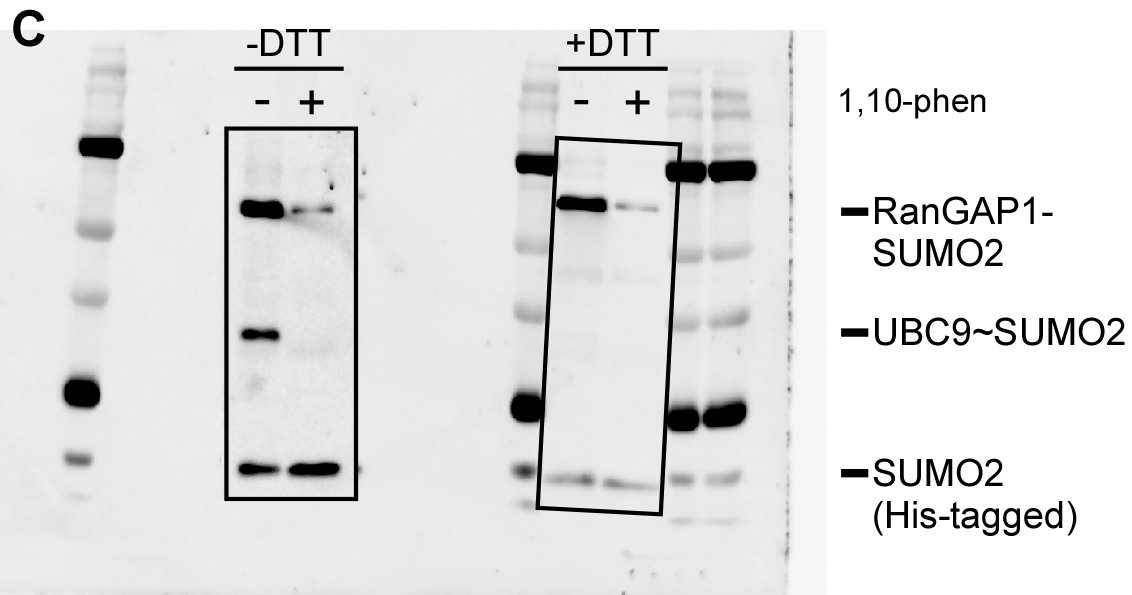

Supplement: Supplementary file 4 — Source Data Fig. 2 [file 44319_2023_10_MOESM4_ESM.zip › Figure 2/2C.tif]

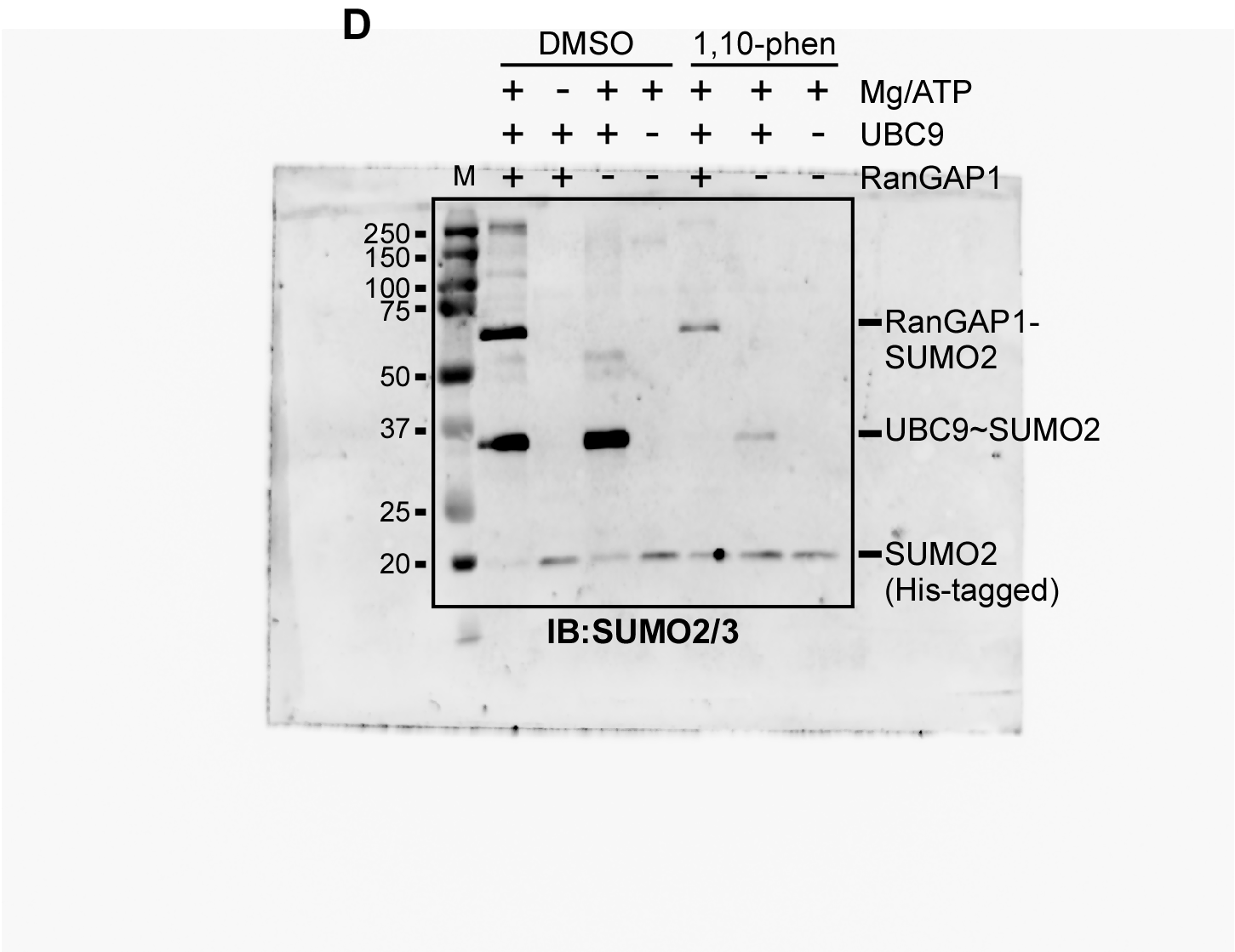

Supplement: Supplementary file 4 — Source Data Fig. 2 [file 44319_2023_10_MOESM4_ESM.zip › Figure 2/2D.tif]

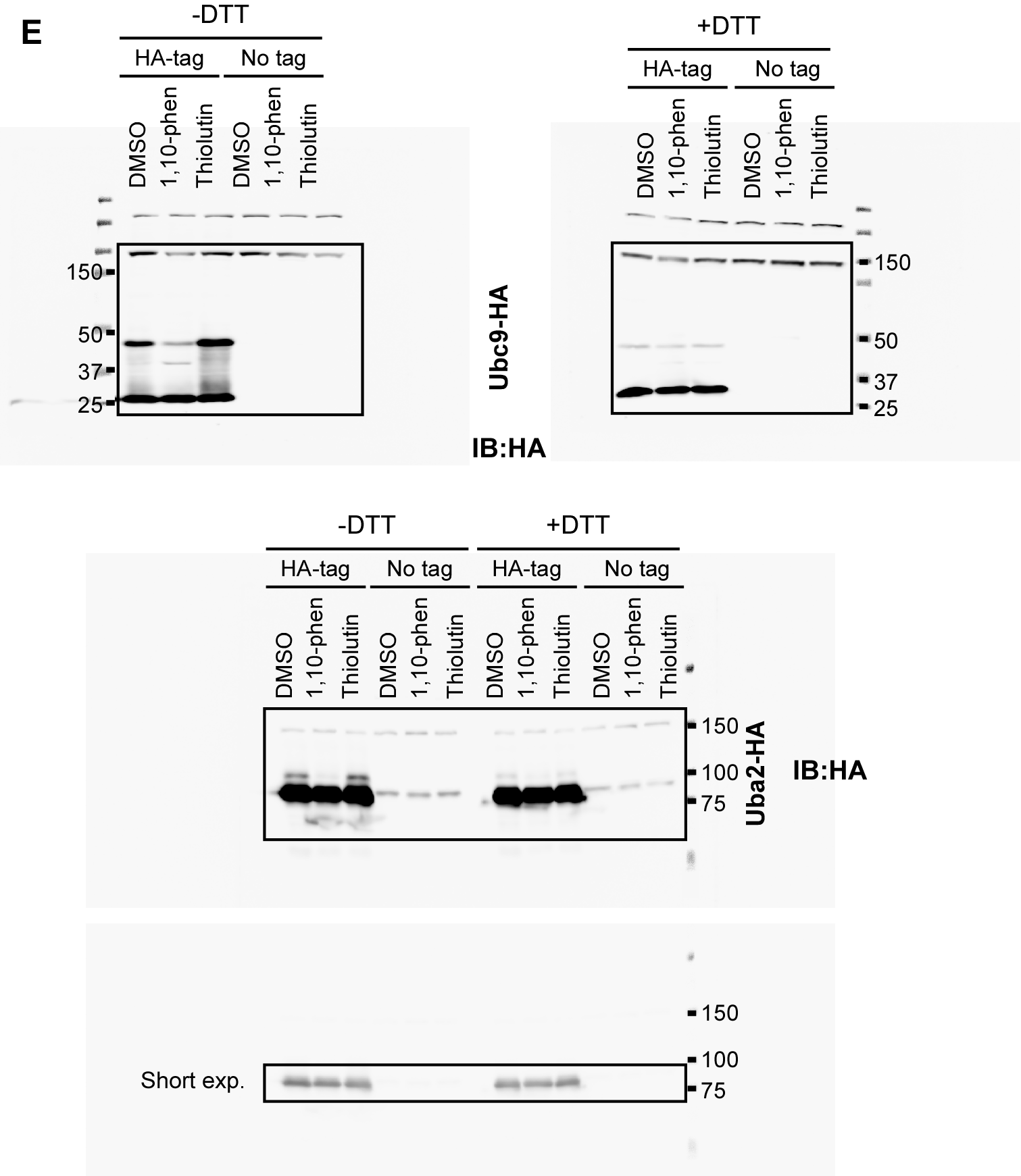

Supplement: Supplementary file 4 — Source Data Fig. 2 [file 44319_2023_10_MOESM4_ESM.zip › Figure 2/2E.tif]

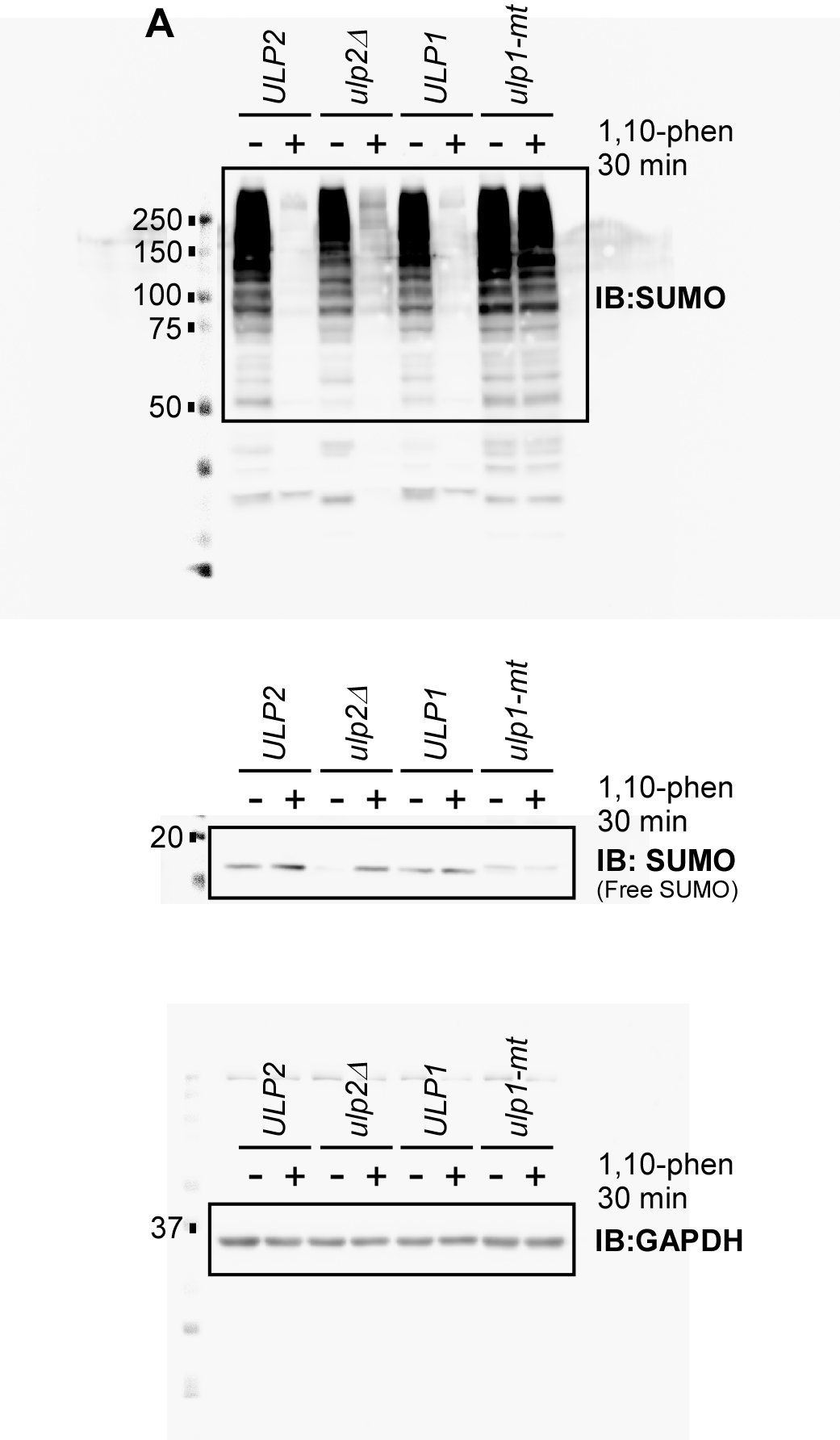

Supplement: Supplementary file 5 — Source Data Fig. 3 [file 44319_2023_10_MOESM5_ESM.zip › Figure 3/3A.tif]

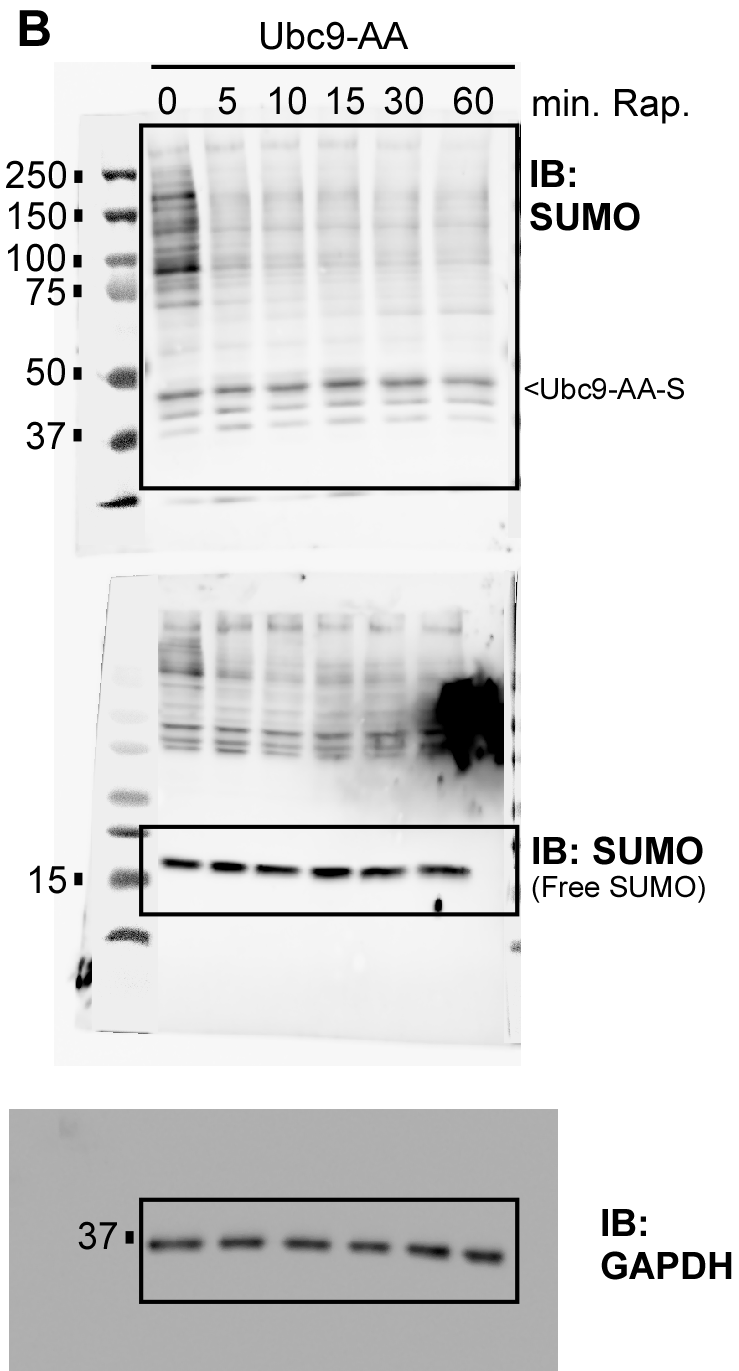

Supplement: Supplementary file 5 — Source Data Fig. 3 [file 44319_2023_10_MOESM5_ESM.zip › Figure 3/3B.tif]

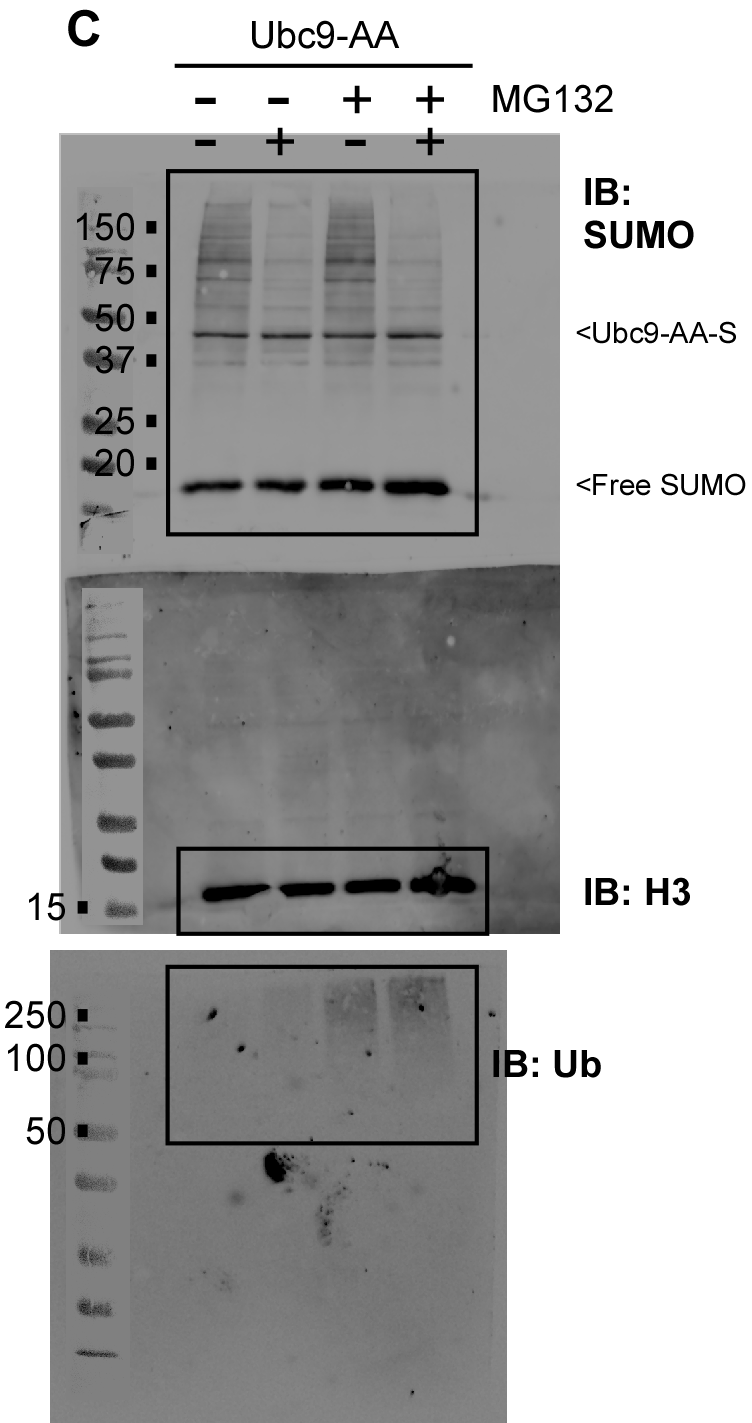

Supplement: Supplementary file 5 — Source Data Fig. 3 [file 44319_2023_10_MOESM5_ESM.zip › Figure 3/3C.tif]

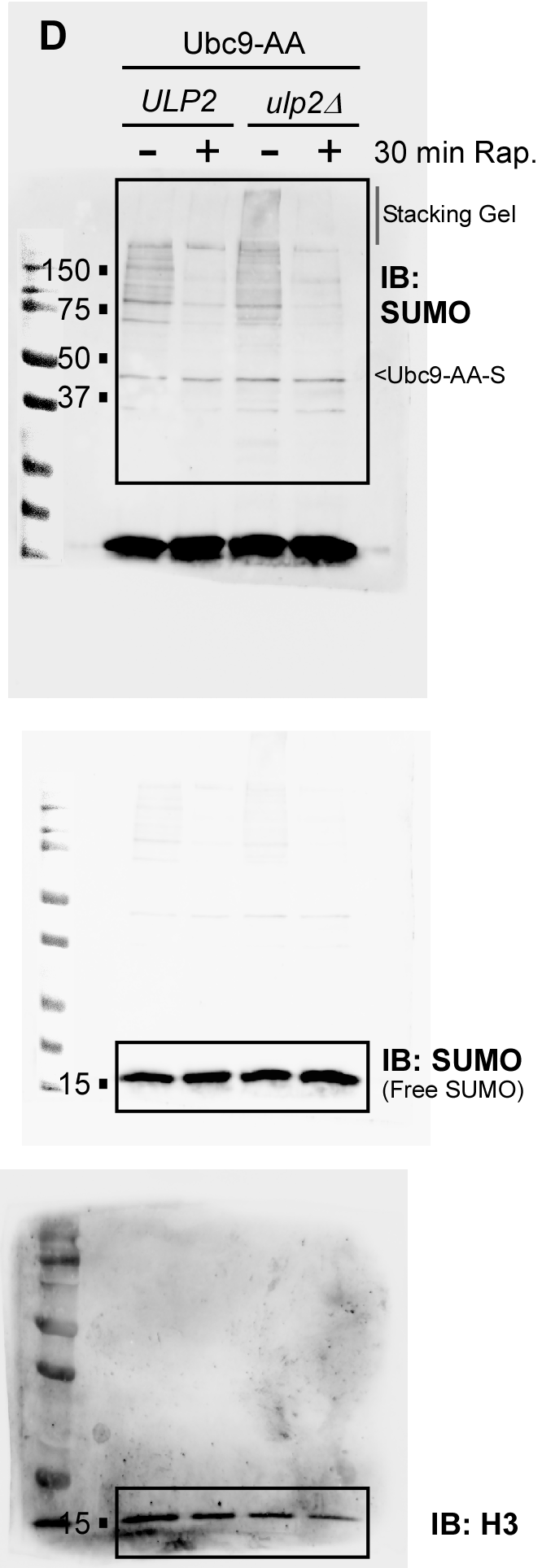

Supplement: Supplementary file 5 — Source Data Fig. 3 [file 44319_2023_10_MOESM5_ESM.zip › Figure 3/3D.tif]

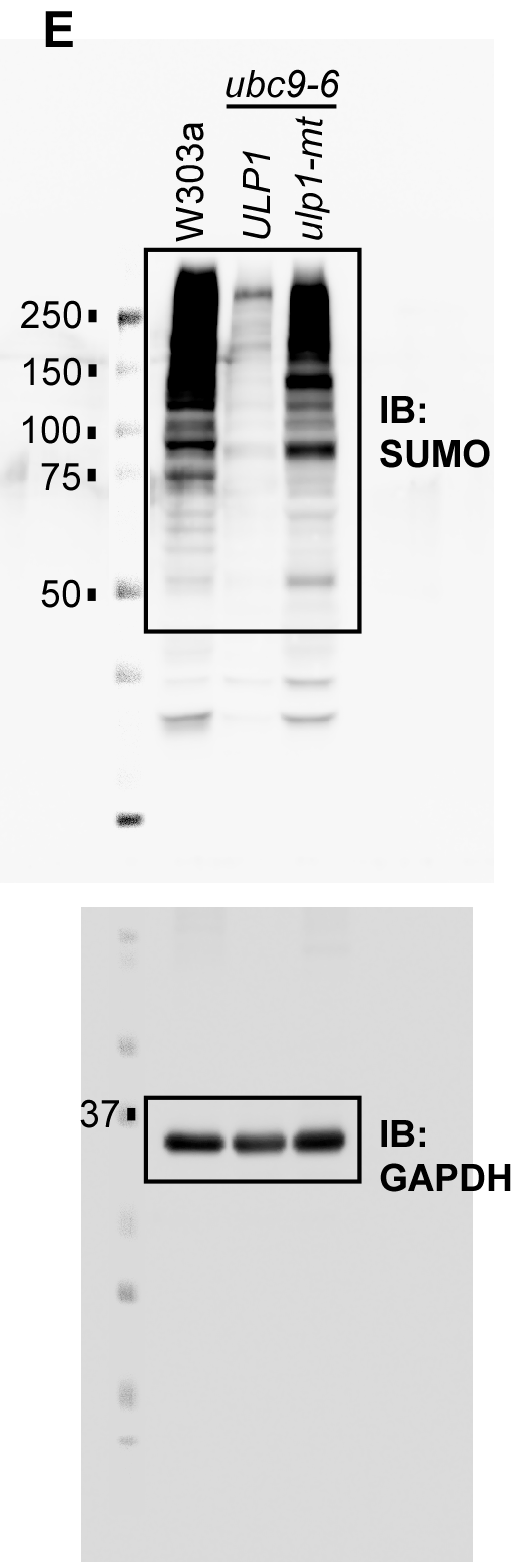

Supplement: Supplementary file 5 — Source Data Fig. 3 [file 44319_2023_10_MOESM5_ESM.zip › Figure 3/3E.tif]

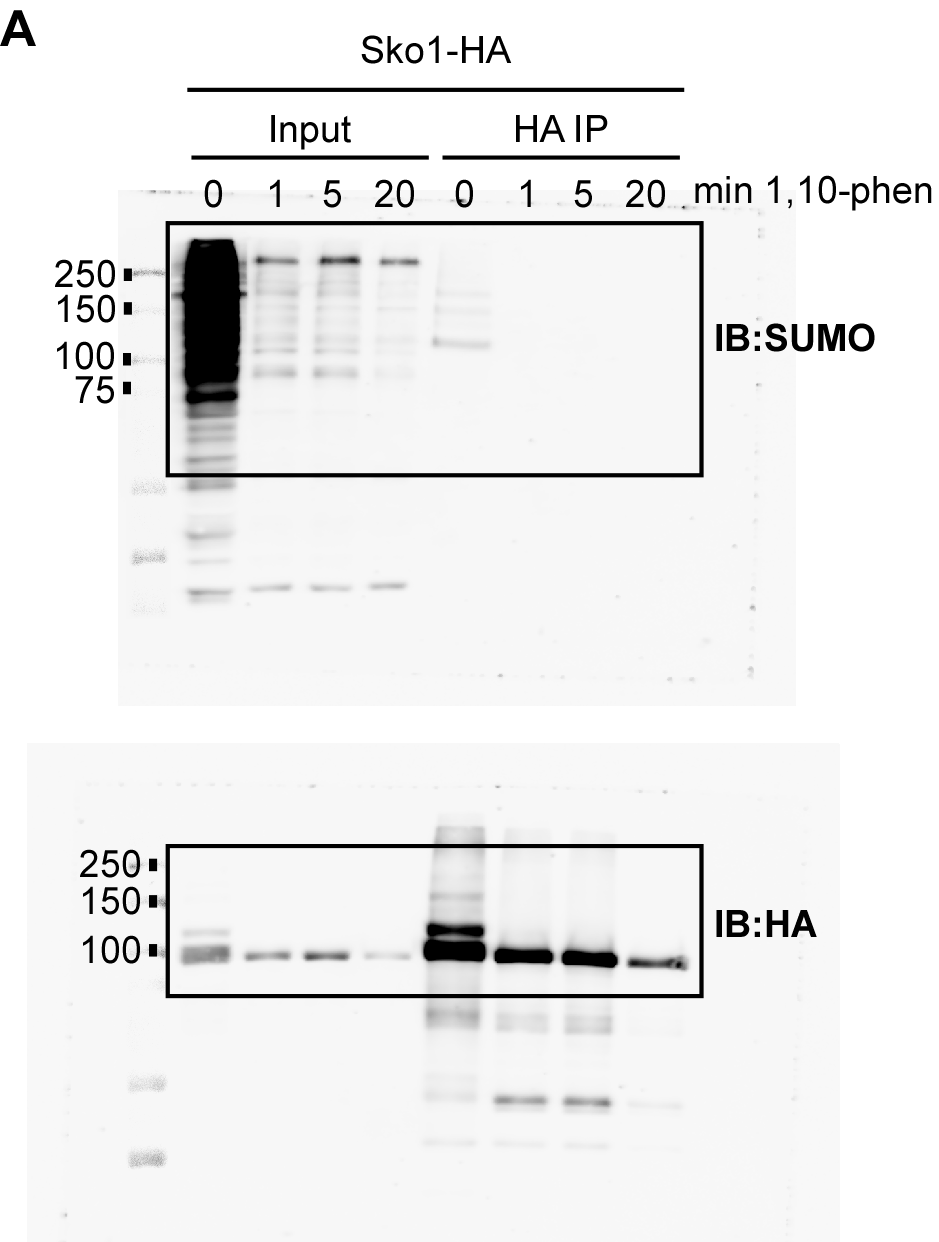

Supplement: Supplementary file 6 — Source Data Fig. 4 [file 44319_2023_10_MOESM6_ESM.zip › Figure 4/4A.tif]

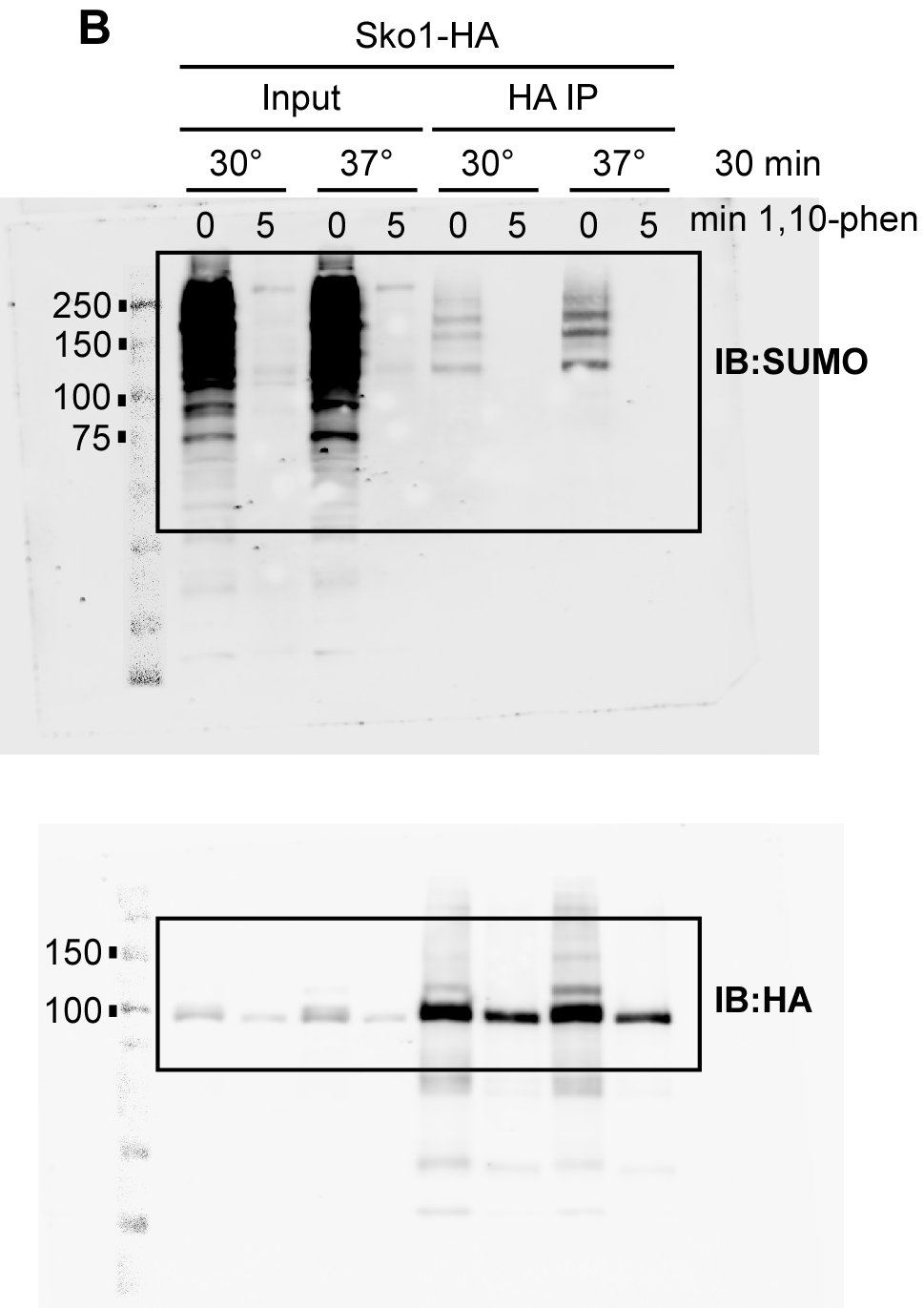

Supplement: Supplementary file 6 — Source Data Fig. 4 [file 44319_2023_10_MOESM6_ESM.zip › Figure 4/4B.tif]

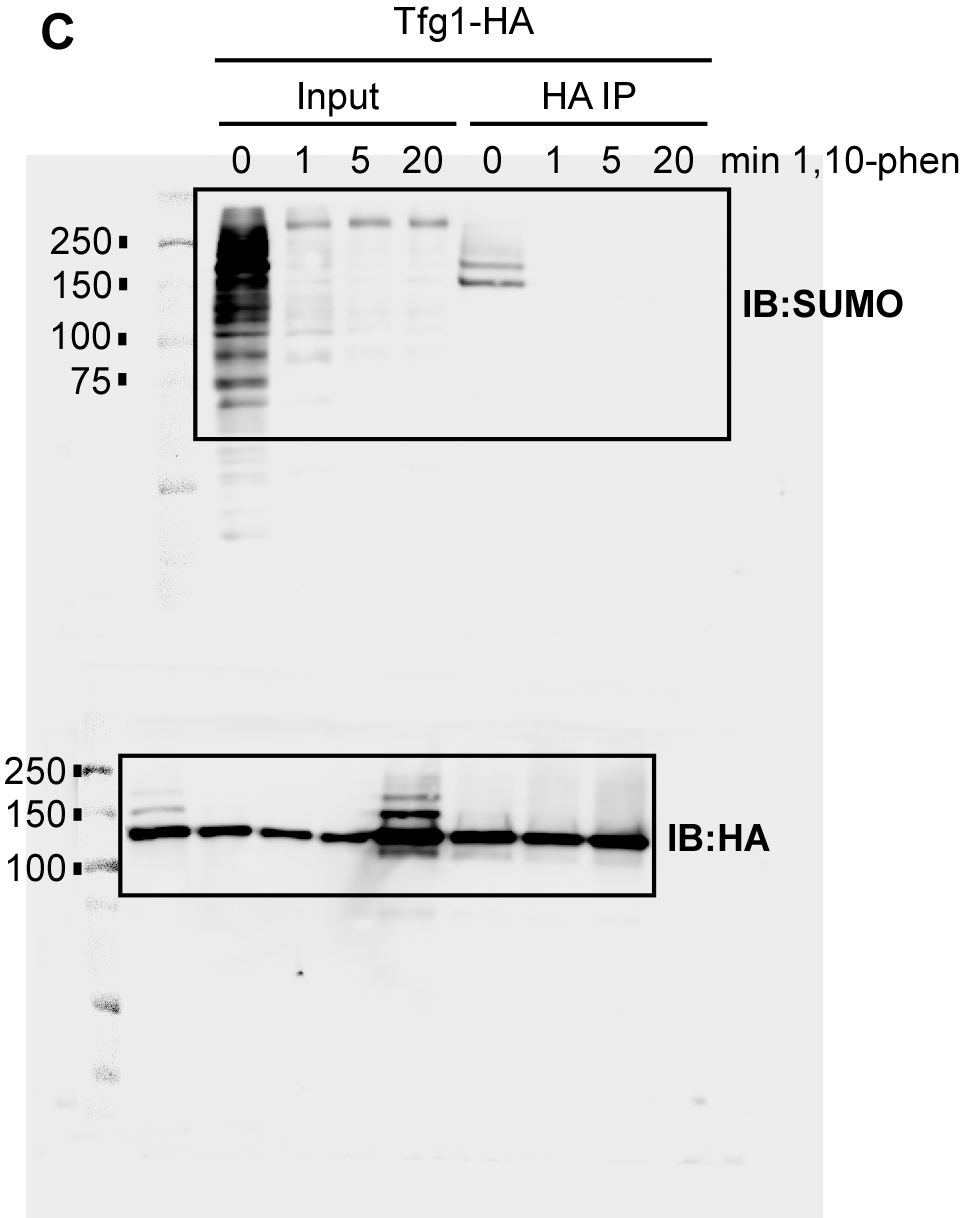

Supplement: Supplementary file 6 — Source Data Fig. 4 [file 44319_2023_10_MOESM6_ESM.zip › Figure 4/4C.tif]

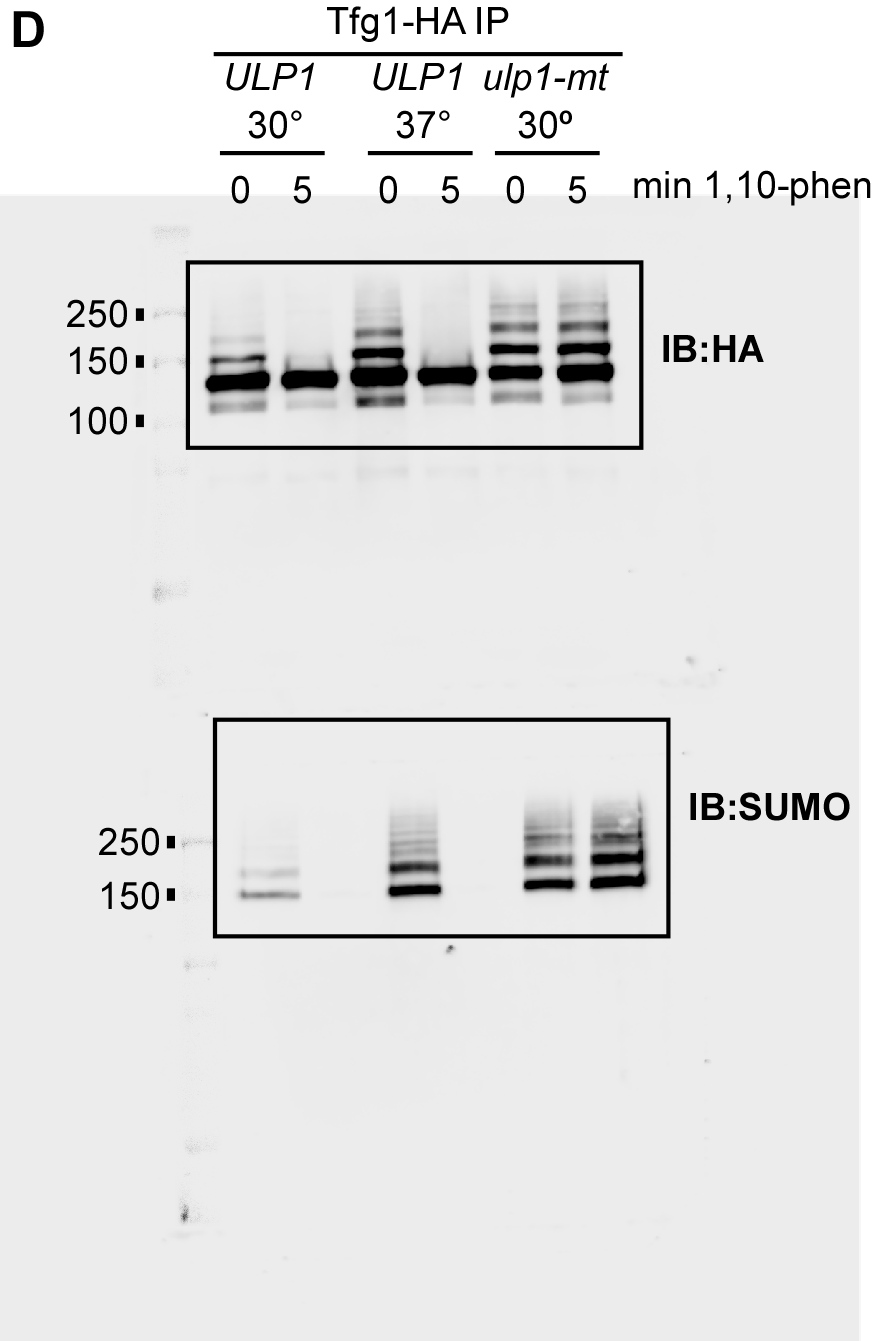

Supplement: Supplementary file 6 — Source Data Fig. 4 [file 44319_2023_10_MOESM6_ESM.zip › Figure 4/4D.tif]

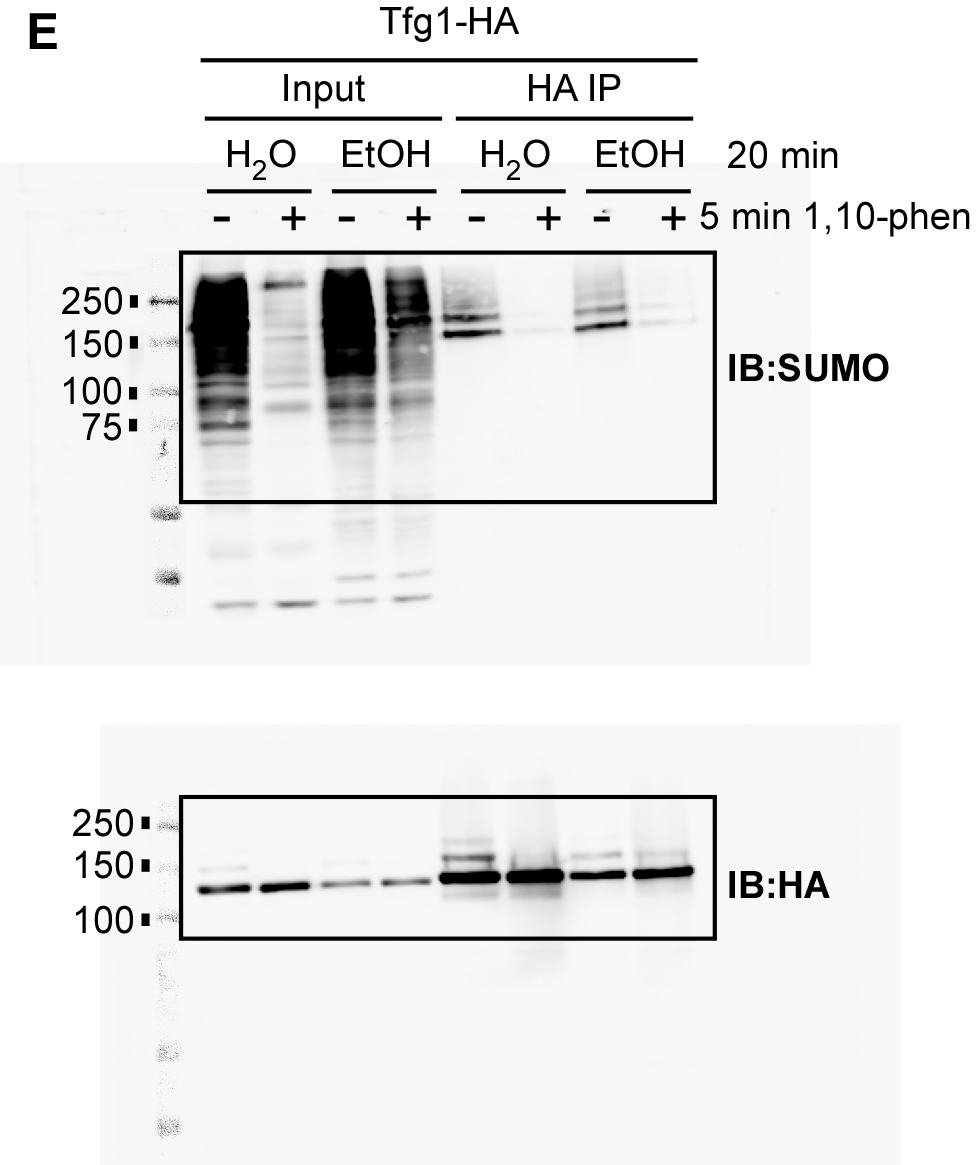

Supplement: Supplementary file 6 — Source Data Fig. 4 [file 44319_2023_10_MOESM6_ESM.zip › Figure 4/4E.tif]

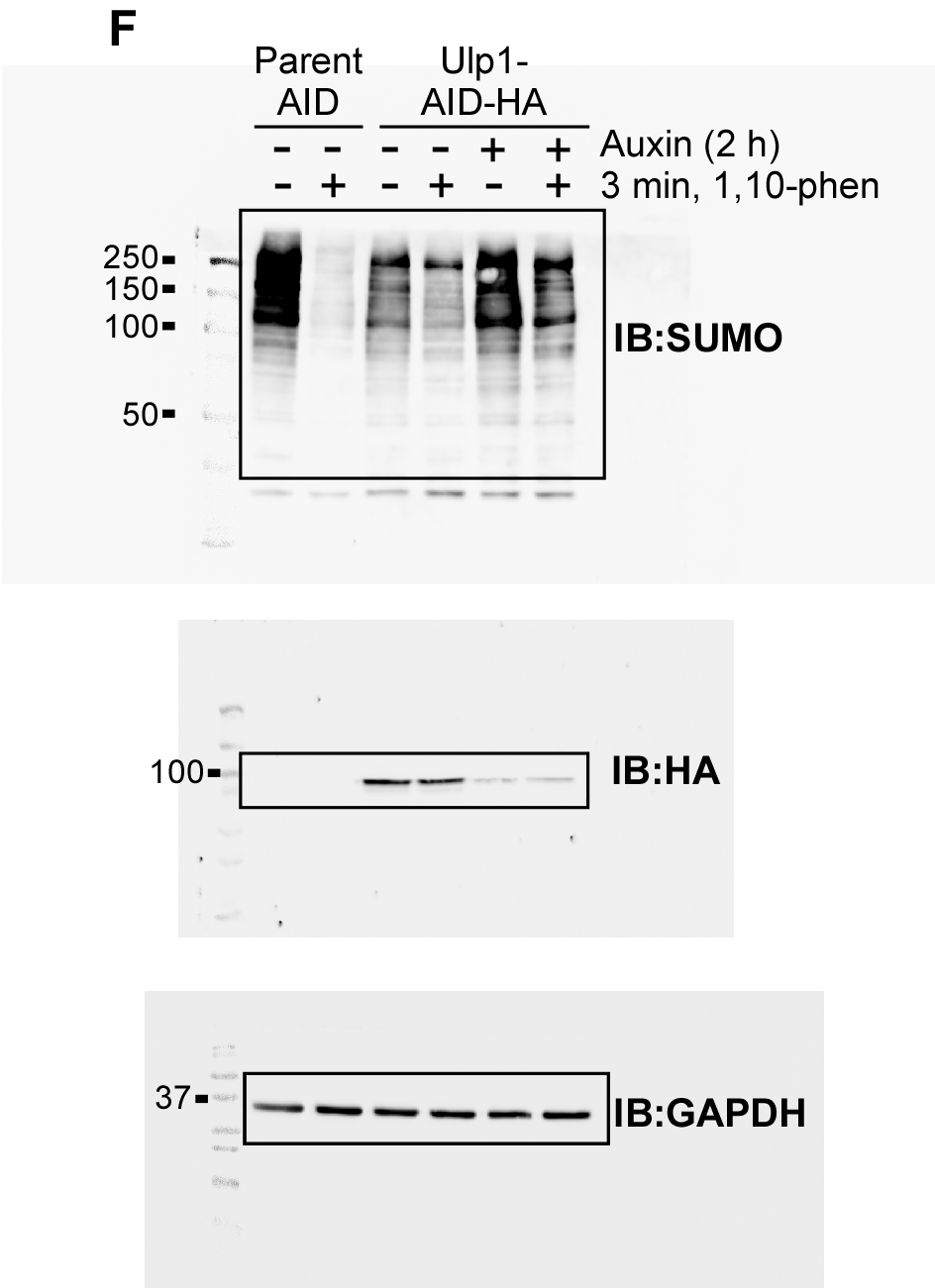

Supplement: Supplementary file 6 — Source Data Fig. 4 [file 44319_2023_10_MOESM6_ESM.zip › Figure 4/4F.tif]
